# STRESS, NEGATIVE AFFECT, AND NEUROTICISM ACROSS THE LIFESPAN

**DOI:** 10.1093/geroni/igae098.1885

**Published:** 2024-12-31

**Authors:** Giselle Ferguson, Samantha Corley, Stacey Scott

**Affiliations:** Union College, Schenectady, New York, United States; Stony Brook University, Stony Brook, New York, United States; Stony Brook University, Stony Brook, New York, United States

## Abstract

Emotional regulation and appraisal skills in response to stressors are thought to accumulate across the lifespan. Work with age-heterogeneous samples found that despite similar initial levels of stressor related Negative Affect (NA), Older Adults (OA) exhibited lower levels of NA over time perhaps due to enhanced emotional recovery. Prior work focused on OA has shown longitudinal increases in within-person NA but a lack of age related stressor-NA change cross-sectionally. We aimed to examine if the relationship between reported momentary stressor occurrence and NA is moderated by baseline neuroticism among an age-heterogenous adult sample and if there are cross-sectional age differences in this relationship. During April-October 2023, participants (N=207) 18-82 years old were recruited from a national online research panel. Participants completed surveys of trait neuroticism then completed seven days of Ecological Momentary Assessments of stressors and NA three times each day. Within persons, negative affect was significantly higher at observations following stressors than non-stressor observations (B=23.01, SE=3.12, p<0.001). Between persons, individuals who reported more frequent stressors across the study also had higher average NA (B=21.90, SE=5.99, p=0.0003). Individual differences in neither neuroticism nor age were significantly related to average NA. Unexpectedly, neither neuroticism nor age moderated the momentary association between stressors and NA. Contrary to our predictions, individual differences in stressor-related increases in NA are not explained by either cross-sectional age differences nor neuroticism. Given the similarity in responses across adult age, future research should examine the role other individual and contextual differences, including stressor type, play in this relationship.

